# Diagnostic yield of routine daily blood culture in patients on veno-arterial extracorporeal membrane oxygenation

**DOI:** 10.1186/s13054-021-03658-7

**Published:** 2021-07-08

**Authors:** Quentin de Roux, Marie Renaudier, Wulfran Bougouin, Johanna Boccara, Vincent Fihman, Raphaël Lepeule, Chamsedine Cherait, Antonio Fiore, François Hemery, Jean-Winoc Decousser, Olivier Langeron, Nicolas Mongardon

**Affiliations:** 1 Service d’Anesthésie-Réanimations Chirurgicales, DMU CARE, DHU A-TVB, Assistance Publique-Hôpitaux de Paris (AP-HP), Hôpitaux Universitaires Henri Mondor, 1 rue Gustave Eiffel, 94000 Créteil, France; 2 Réanimation Polyvalente, Hôpital Privé Jacques Cartier, 91300 Massy, France; 3 Département de prévention, diagnostic et traitement des infections, Assistance Publique des Hôpitaux de Paris (APHP), Hôpitaux Universitaires Henri Mondor, 94010 Créteil, France; 4 Unité transversale de traitement des infections, Assistance Publique des Hôpitaux de Paris (APHP), Hôpitaux Universitaires Henri Mondor, 94010 Créteil, France; 5 Service de chirurgie cardiaque, Assistance Publique des Hôpitaux de Paris (APHP), Hôpitaux Universitaires Henri Mondor, 94010 Créteil, France; 6 Département d’information médicale, Assistance Publique des Hôpitaux de Paris (APHP), Hôpitaux Universitaires Henri Mondor, 94010 Créteil, France; 7 Faculté de Santé, Univ Paris Est Créteil, 94010 Créteil, France; 8 U955-IMRB, Equipe 03 “Pharmacologie et Technologies pour les Maladies Cardiovasculaires (PROTECT)”, Inserm, Univ Paris Est Créteil (UPEC), Ecole Nationale Vétérinaire d’Alfort (EnVA), 94700 Maisons-Alfort, France

**Keywords:** Extracorporeal membrane oxygenation, Cardiogenic shock, Blood culture, Bloodstream infection, Contamination

## Abstract

**Background:**

Bloodstream infections (BSIs) are frequent on veno-arterial extracorporeal membrane oxygenation (V-A ECMO). Performing routine blood cultures (BCs) may identify early paucisymptomatic BSIs. We investigated the contribution of systematic daily BCs to detect BSIs on V-A ECMO.

**Methods:**

This was a retrospective study including all adult patients requiring V-A ECMO and surviving more than 24 h. Our protocol included routine daily BCs, from V-A ECMO insertion up to 5 days after withdrawal; other BCs were performed on-demand.

**Results:**

On the 150 V-A ECMO included, 2146 BCs were performed (1162 routine and 984 on-demand BCs); 190 (9%) were positive, including 68 contaminants. Fifty-one (4%) routine BCs revealed BSIs; meanwhile, 71 (7%) on-demand BCs revealed BSIs (*p* = 0.005). Performing routine BCs was negatively associated with BSIs diagnosis (OR 0.55, 95% CI [0.38; 0.81], *p* = 0.002). However, 16 (31%) BSIs diagnosed by routine BCs would have been missed by on-demand BCs. Independent variables for BSIs diagnosis after routine BCs were: V-A ECMO for cardiac graft failure (OR 2.43, 95% CI [1.20; 4.92], *p* = 0.013) and sampling with on-going antimicrobial therapy (OR 2.15, 95% CI [1.08; 4.27], *p* = 0.029) or renal replacement therapy (OR 2.05, 95% CI [1.10; 3.81], *p* = 0.008). Without these three conditions, only two BSIs diagnosed with routine BCs would have been missed by on-demand BCs sampling.

**Conclusions:**

Although routine daily BCs are less effective than on-demand BCs and expose to contamination and inappropriate antimicrobial therapy, a policy restricted to on-demand BCs would omit a significant proportion of BSIs. This argues for a tailored approach to routine daily BCs on V-A ECMO, based on risk factors for positivity.

## Introduction

Veno-arterial extracorporeal membrane oxygenation (V-A ECMO) is increasingly used to support various causes of refractory shock [1]. Although it is life-saving support, it generates new issues and side effects. Emergent management, invasive procedures and devices, and intensive care unit (ICU)-acquired immunodepression increase infectious risk. Indeed, half of patients will further develop sepsis on V-A ECMO support [2]. Infectious complications are associated with an around 50% increase of mortality [3].

The diagnosis of sepsis in adult population supported by V-A ECMO is challenging. Body temperature cannot be interpreted because of blood exposure to heat exchanger. White blood cell count and common biomarkers lack of specificity in the setting of cardiogenic shock both on medical treatment and on V-A ECMO [4, 5]. To overcome diagnosis, routine blood cultures (BCs) are proposed [6, 7]. In a survey of the Extracorporeal Life Support Organization (ELSO), one-third of the American centers performed daily routine BCs [8]. However, this practice is still debated and has never been evaluated on V-A ECMO [9, 10]. According to the low incidence of poorly symptomatic BSIs and the high rate of contaminants leading to unnecessary antimicrobial therapy, we hypothesized that systematic BCs have a lowest interest than clinically guided BCs.

Beyond determining incidence, risk factors, and consequences of positive BCs, the aim of this study was to determine the respective contribution of routine versus on-demand BCs in a population of adult patients requiring V-A ECMO for refractory cardiogenic shock.

## Patients and methods

### Study design and population

All adult patients who underwent peripheral V-A ECMO for medical or surgical refractory cardiogenic shock, or for refractory cardiac arrest, in our 15-bed cardiovascular surgical ICU (Henri Mondor Teaching Hospital, Créteil, France) from January 2013 to January 2017 were retrospectively included. Patients who died within 24 h after V-A ECMO implantation were excluded.

### V-A ECMO management

In case of refractory cardiac arrest or refractory cardiogenic shock, femoro-femoral V-A ECMO was inserted through surgical approach. Indications for V-A ECMO assistance followed recommendations for management of shock refractory to fluid optimization and inotrope/vasopressive drugs administration [11]. Withdrawal was performed according to standard recommendations [12].

### Antimicrobial strategy and hygiene practices

Our protocol does not include antimicrobial prophylaxis in case of V-A ECMO implantation in ICU [13]. When the implantation is performed in the operating room in case of immediate cardiopulmonary bypass weaning failure, antibiotic prophylaxis is restricted to the ongoing surgical procedure (repeated dose of cefazolin up to chest skin closure, or a single bolus of vancomycin in case of beta-lactam allergy). Neither digestive decontamination nor systemic antimicrobial prophylaxis is implemented during ICU stay. If sepsis is suspected, an empiric antimicrobial therapy is initiated according to our local protocol and secondarily tailored to the laboratory findings. Daily rounds with microbiologists and infectious diseases specialists are carried out [14].

In case of scheduled cardiac surgery, nasal carriage of *Staphylococcus aureus* is screened the day before surgery and treated by a five-day course of nasal mupirocin in case of positivity, or otherwise stopped.

For body routine care, daily skin washing with chlorhexidine 4% (Hibiscrub®, BCM Limited, Nottingham, UK) is performed during the first seven postoperative days in *Staphylococcus aureus* nasal carriers; other usual hygiene procedures are applied [15]. The sites of cannulations are cleaned with chlorhexidine 2% in alcohol isopropyl 70% (Bactiseptic®, Vesismin S.L., Barcelona, Spain) and covered with occlusive dressings changed each two days. No antiseptic dressing is used. Care bundles for the prevention of ventilator-associated pneumonia follow international recommendations [16].

### Blood culture microbiological samples practices

Our local protocol recommends daily routine BCs, from V-A ECMO implantation up to five days after withdrawal. After decontamination of the skin and lid of bottles with chlorhexidine 2%, 10 mL of blood is sampled in one aerobic and anaerobic bottle from the arterial line [17]. BCs performed between 4 and 7 a.m. were defined as routine BCs; all other BCs were considered as on-demand BCs. BCs results from the day of V-A ECMO implantation up to five days after withdrawal were included. Other microbiological samples were left to the intensivist’s discretion.

### Bloodstream infection definition

As defined by the Centers for Disease Control and Prevention, *Corynebacterium* spp, *Bacillus* spp, *Cutibacterium* spp, coagulase-negative *Staphylococci* (CoNS), viridians group *Streptococci*, *Aerococcus* spp and *Micrococcus* spp were considered as common skin contaminants unless the same bacterial strain was isolated from two separate BCs within 48 h of each other [18, 19]. All other pathogens were considered as BSIs from the first positive BC. BSI was considered as primary when the microorganism isolated in the BC was not clinically related to an infectious source. Otherwise, if the pathogen isolated in the BC corresponded to a pathogen identified from another sterile site, BSI was considered as secondary. Several BCs positive with the same pathogen on consecutive samples or days were considered as multiple positive BCs but belonging to the same single episode of BSI [20].

### Appropriateness of antimicrobial therapy

We considered antimicrobial therapy as appropriate (defined as the use of agents with in vitro activity against the etiologic pathogens) when it was administered for relevant BSI and within 24 h after the final antimicrobial report [21, 22]. Antimicrobial therapy was a posteriori deemed inappropriate when beginning for a single BC positive with a contaminant pathogen.

### Data collection and ethical considerations

Data were collected from patient’s medical files and from the microbiological department database. We collected pre-morbid and demographic conditions, characteristics of V-A ECMO support, clinical and biological parameters, and ICU/hospital course and outcomes. For each BC, body temperature, site of sampling, and sampling conditions were analyzed.

According to the French law, patients were informed of the anonymous data extraction and analysis from medical files [23]. This study was approved by the Comité d’Ethique pour la Recherche en Anesthésie-Réanimation (CERAR, IRB 00010254-2019-027).

### Statistical analysis

Continuous variables were expressed as median and interquartile range [25–75%] or mean (standard deviation), as appropriate. Categorical variables were expressed as proportions. Firstly, patients were compared according to occurrence of at least one BSI episode using *χ*^2^ test for categorical variables and Student *t*-test, Mann–Whitney, or Kruskal–Wallis test for continuous variables, as appropriate. Secondly, BCs were compared (BSI vs no BSI) using *χ*^2^ test for categorical variables and Student t-test, Mann–Whitney, or Kruskal–Wallis test for continuous variables, considering each BC as an independent unit. Finally, variables associated with BSI in univariate analysis (with *p* < 0.15) were included in a multivariable logistic regression to identify factors independently associated with BSI. After multivariable logistic regression, we assessed the test performance (sensitivity, specificity, positive predictive value, and negative predictive value) of risk factors identified in the multivariable model. A sensitivity analysis restricted to routine BCs was performed. As a sensitivity analysis, we performed a multilevel logistic modeling, considering blood samples as level 1 (with “level 1 variables” including collection on V-A ECMO, routine sampling, ongoing microbial therapy, and ongoing RRT) and patients as level 2 (with BMI, KDIGO, lactate level, and bilirubin level as “level 2 variables”). All tests were two-sided, with *p* < 0.05 considered statistically significant. STATA/SE 15.0 software (College Station, TX, USA) was used for statistical analysis.

## Results

**Fig. 1:**
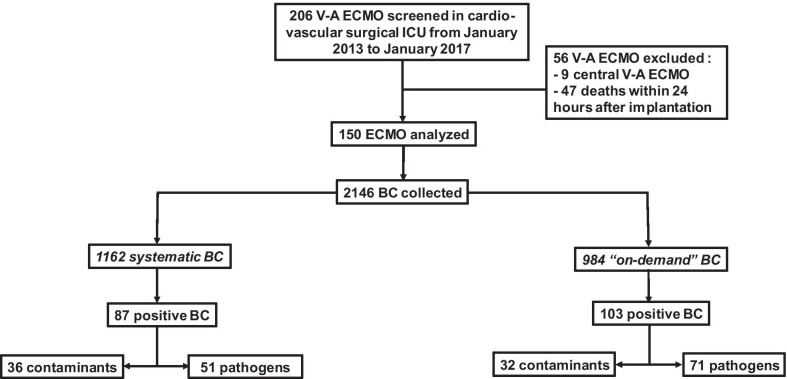
Flow chart

During the 4-year study period, 206 consecutive VA-ECMO were inserted for refractory cardiogenic shock or cardiac arrest. After exclusion of 56 V-A ECMO (9 patients with central V-A ECMO and 47 deaths within 24 h after implantation), 150 V-A ECMO were analyzed (Fig. 1). Patients who received two V-A ECMO during hospitalization (*n* = 3) were considered as independent cases.

### Baseline patients’ characteristics

Median age was 58 [48–69] years. Comorbidities are reported in Table 1. V-A ECMO indication was mainly acute medical heart failure (*n* = 85; including 39 refractory cardiac arrest); post-cardiotomy shock concerned 65 V-A ECMO indications (including 14 primary graft failure after heart transplantation). All patients except one were mechanically ventilated. Baseline patients’ characteristics did not differ regarding BSIs onset, except for patients with V-A ECMO for graft failure for whom BSIs were significantly more frequent, and for lactate level at admission, which was higher in patients without BSIs.

**Table 1:** Baseline patients characteristics

Characteristics	All V-A ECMO (*n* = 150)	Patient without BSI (*n* = 100)	Patient with BSI (*n* = 50)	*p*
**Age (years)**	58 (16)	59 (16)	55 (15)	0.14
**Male gender**	104 (69)	68 (68)	36 (72)	0.62
**BMI (kg/m)**^**2**^	25.4 (22.8–29.0)	24.7 (22.8–28.7)	27.1 (22.6–29.4)	0.13
**Comorbidities**				
COPD	10 (7)	7 (7)	3 (6)	0.82
Cirrhosis	3 (2)	1 (1)	2 (4)	0.22
Long-term corticosteroid	7 (5)	4 (4)	3 (6)	0.58
Diabetes	36 (24)	27 (27)	9 (18)	0.22
**V-A ECMO for post-cardiotomy shock**	65 (43)	43 (43)	22 (44)	0.91
Including primary graft failure after heart transplantation	14 (21)	5 (11)	9 (41)	0.01
**V-A ECMO for acute heart failure**	85 (57)	57 (57)	28 (56)	0.91
Including refractory cardiac arrest	39 (26)	12 (24)	27 (27)	0.69
**Percutaneous V-A ECMO insertion**	9 (6)	8 (8)	1 (2)	0.27
**Intra-aortic balloon pump**	26 (17)	20 (20)	6 (12)	0.22
**Lactate level at day 0 (mmol/L)**	5.2 (3.0–9.1)	6.1 (3.7–10.3)	3.8 (2.2–8.3)	0.003
**Creatinine level at day 0 (µmol/L)**	159 (98)	154 (93)	169 (109)	0.39
**SAPS II**	54 (38–70)	56 (40–74)	50 (37–66)	0.19
**Admission SOFA score**	13 (11–15)	14 (12–16)	12 (11–15)	0.10
**Vasoactive–inotropic score**	70 (34–139)	68 (36–130)	73 (29–141)	0.91

Data are expressed as median (interquartile 25–75) or number (percentage), as appropriate

V-A ECMO, veno-arterial extracorporeal membrane oxygenation; BSI, bloodstream infection; BMI, body mass index; COPD, chronic obstructive pulmonary disease; SAPS II, Simplified Acute Physiology Score 2; SOFA, Sepsis-related Organ Failure Assessment

### Clinical characteristics and onset of bloodstream infection during ECMO course

**Fig. 2:**
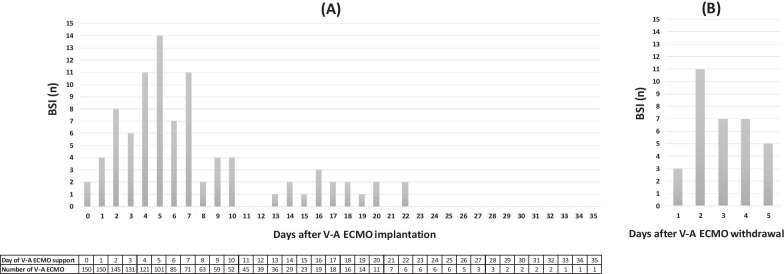
Bloodstream infections per day, according to time elapsed after V-A ECMO implantation (panel **A**) or withdrawal (panel **B**)

Median duration of V-A ECMO support was 7 [5–13] days, representing a total of 1422 ECMO-days. BSIs occurred in 50 (33%) patients, with increased incidence during the first week and after withdrawal (Fig. 2). Pathogens identified in BSIs were reported in Table 2. V-A ECMO support duration was significantly longer in case of BSIs (11 versus 6 days, *p* = 0.0002). Similarly, mechanical ventilation support duration for patients with BSIs was 6 days longer (*p* = 0.02).

**Table 2:** Pathogens responsible for bloodstream infection on V-A ECMO

Pathogen	Overall	Within day 0–day 7	Within day 7 withdrawal	After ECMO withdrawal
*Klebsiella pneumoniae*	24	14	8	2
*Enterobacter aerogenes*	18	2	3	13
*Proteus mirabilis*	15	8	1	6
*Enterobacter cloacae*	13	10	2	1
*Escherichia coli*	11	6	2	3
*Pseudomonas aeruginosa*	13	3	0	10
*Yeast (including Candida spp.)*	6	0	5	1
*Enterococcus faecalis*	6	5	0	1
*Hafnia alvei*	5	5	0	0
*Bacteroides fragilis*	3	1	1	1
*Klebsiella oxytoca*	3	0	2	1
*Pantoea spp.*	3	3	0	0
*Serratia marcescens*	2	0	0	2
*Staphylococcus aureus*	2	2	0	0
*Citrobacter braakii*	1	1	0	0
*Enterococcus faecium*	1	0	0	1
*Haemophilus influenzae*	1	1	0	0
*Lactobacillus casei*	1	1	0	0
*Neisseria spp.*	1	1	0	0
*Leuconostoc spp.*	1	1	0	0
*Morganella morganii*	1	1	0	0
*Stenotrophomonas maltophilia*	1	1	0	0

Total numbers of pathogens differ from number of BSI due to polymicrobial BSI

Overall, ICU mortality reached 56% without significant difference between patients with or without BSIs. ICU and hospital lengths of stay were significantly longer in case of BSIs (Table 3).

**Table 3:** Clinical characteristics during ECMO course

	All V-A ECMO (*n* = 150)	Patient without BSI (*n* = 100)	Patient with BSI (*n* = 50)	*p*
**ECMO implantation in the operative room**	81 (54)	55 (55)	26 (52)	0.73
**V-A ECMO support duration (days)**	7 (5–13)	6 (5–11)	11 (6–16)	0.0002
**Total red blood cell unit transfusion during V-A ECMO support**	13 (9)	13 (9)	13 (9)	0.83
**Acute mesenteric ischemia during V-A ECMO support**	14 (9)	8 (8)	6 (12)	0.43
**Total duration of mechanical ventilation (days)**	14 (6–27)	12 (5.5–25)	18 (10–30)	0.02
**ICU mortality**	84 (56)	54 (54)	30 (60)	0.49
**ICU length of stay (days)**	19 (10–32)	17 (9–26)	23 (14–38)	0.02
**Hospital length of stay (days)**	24 (14–38)	21 (10–37)	31 (17–41)	0.01

Data are expressed as median (interquartile 25–75) or number (percentage), as appropriate

V-A ECMO, veno-arterial extracorporeal membrane oxygenation; BSI, bloodstream infection; ICU, intensive care unit

### Blood cultures practices and results

Overall, 2146 BCs were collected (including 363 BCs up to five days after V-A ECMO withdrawal), corresponding to a mean of 1.5 BCs per day. Seventy percent of the 1422 days of V-A ECMO had been subject to one routine BC sampling. Overall, 190 BCs (9%) were positive: 116 with bacteria and 6 with yeasts, *i.e.,* 122 BSIs, after exclusion of 68 contaminants. Forty-five (36%) BSIs were considered as primary. During V-A ECMO course, 49 different episodes of BSIs were observed, i.e., a BSI rate of 34.5 cases/1.000 days of V-A ECMO support.

Regarding sampling categorization, on 1162 routine BCs performed, 51 were positive for non-contaminants pathogens (4%). Conversely, 984 on-demand BCs were sampled and 71 were positive for non-contaminants pathogens (7%) (*p* = 0.005). On the 68 total BCs positive for contaminants, 10 (15%) led to an inappropriate antimicrobial therapy; this rate did not differ whether contaminant was isolated from routine BCs (*n* = 5) or on-demand BCs (*n* = 5) (13 vs. 14%, *p* = 0.85).

Focusing on routine BCs, 16 (31%) BSIs from routine BCs would not have been diagnosed by on-demand BCs, i.e., patients had no on-demand BCs.

### Blood culture characteristics variables associated with bloodstream infection

Considering each BC as an independent event, clinical and BC characteristics during V-A ECMO course are summarized in Table 4. Body temperature at the time of BC sampling was not different in case of BSI. Whereas BCs were preferably drawn from arterial line (70%), BSI was more observed when BCs were retrieved from central venous line or direct venipuncture (*p* < 0.001).

**Table 4:** Blood culture characteristics

	All BC (*n* = 2146)	No BSI (*n* = 2024)	BSI (*n* = 122)	*p*
**Age (years)**	57 (16)	57 (16)	54 (16)	0.04
**BMI (kg/m)**^**2**^	26.1 (4.6)	26.1 (4.6)	26.8 (4.6)	0.12
**Indications for V-A ECMO**				< 0.001
Acute heart failure	763 (36)	720 (36)	43 (35)	
Refractory cardiac arrest	466 (22)	446 (22)	20 (16)	
Post-cardiotomy shock	722 (34)	691 (34)	31 (25)	
Primary graft failure	195 (9)	167 (8)	28 (23)	
**Percutaneous V-A ECMO insertion**	119 (6)	111 (9)	8 (1)	< 0.001
**Hemoglobin at day 0 (g/dL)**	10.0 (2.4)	9.9 (2.3)	10,2 (2.7)	0.23
**White blood cell count at day 0 (G/L)**	14.2 (6.8)	14.2 (6.8)	14.5 (7.5)	0.69
**Total bilirubin level at day 0 (µmol/L)**	33 (46)	32 (43)	46 (85)	0.002
**Lactate level at day 0 (mmol/L)**	6.0 (4.1)	6.1 (4.2)	4.5 (3.4)	< 0.0001
**KDIGO stage **				0.026
0	813 (38)	781 (39)	32 (26)	
1	634 (30)	587 (29)	47 (39)	
2	276 (13)	255 (13)	21 (17)	
3	423 (20)	401 (20)	22 (18)	
**Timing between V-A ECMO implantation and BC sampling**				0.001
< 7 days	1124 (52)	1069 (53)	55 (45)	
7–15 days	733 (34)	696 (34)	37 (30)	
> 15 days	289 (13)	259 (13)	30 (25)	
**BC sampling on V-A ECMO**	1783 (83)	1694 (84)	89 (73)	0.002
**Routine BC**	1162 (54)	1111 (55)	51 (42)	0.005
**Body temperature**	36.8 (36.4–37.3)	36.8 (36.4–37.3)	36.9 (36.4–37.3)	0.57
≥ 38.3 °C	156 (7)	150 (7)	6 (5)	0.30
**BC sampling site**				< 0.001
Arterial line	1705 (82)	1629 (83)	76 (66)	
Central venous catheter	210 (10)	186 (9)	24 (21)	
Peripheral venipuncture	161 (8)	146 (7)	15 (13)	
**Ongoing antimicrobial therapy**	1318 (61)	1231 (61)	87 (72)	0.02
**Ongoing RRT**	582 (27)	527 (26)	55 (45)	< 0.001

Data are expressed as median [interquartile 25–75] or number (percentage), as appropriate

V-A ECMO, veno-arterial extracorporeal membrane oxygenation; BC, blood culture; BMI, body mass index; BSI, bloodstream infection; KDIGO, Kidney Disease Improving Global Outcomes; RRT, renal replacement therapy

In multivariate analysis considering all BCs (Table 5), independent risk factors associated with BSIs were: BMI (OR 1.1, 95% CI [1.0; 1.1], *p* = 0.007), lactate level at admission (OR 0.90, 95% CI [0.85; 0.95], *p* < 0.001), bilirubin level at admission (OR 1.00, 95% CI [1.00; 1.01], *p* = 0.019), BCs collected on V-A ECMO (OR 0.52, 95% CI [0.34; 0.81], *p* = 0.004), BCs collected with ongoing antimicrobial therapy (OR 1.56, 95% CI [1.03; 2.35], *p* = 0.037), and BCs collected with ongoing renal replacement therapy (OR 2.76, 95% CI [1.86; 4.09], *p* < 0.001). In addition, performing routine BC was negatively associated with BSI diagnosis (OR 0.55, 95% CI [0.38; 0.81], *p* = 0.002). Sensitivity analysis with multilevel model adjusted found consistent results (OR of routine BC for BSI = 0.44, 95% CI [0.28; 0.67], *p* < 0.001).

**Table 5:** Multivariate analysis of independent variables of probability of blood culture to diagnose bloodstream infection

BSI characteristics	OR [95% CI]	*p*
**BMI (kg/m)**^**2**^	1.06 [1.01; 1.11]	0.007
**KDIGO stage **		
1	1.95 [1.19; 3.17]	0.007
2	1.64 [0.91; 2.95]	0.101
3	1.36 [0.77; 2.40]	0.285
**Lactate level at day 0 (mmol/L)**	0.90 [0.85; 0.95]	< 0.001
**Total bilirubin level at day 0 (µmol/L)**	1.00 [1.00; 1.01]	0.019
**Collection on V-A ECMO**	0.52 [0.34; 0.81]	0.004
**Routine sampling**	0.55 [0.38; 0.81]	0.002
**On-going antimicrobial therapy**	1.56 [1.03; 2.35]	0.037
**On-going RRT**	2.76 [1.86; 4.09]	< 0.001

V-A ECMO, veno-arterial extracorporeal membrane oxygenation; BSI, bloodstream infection; BMI, body mass index; KDIGO, Kidney Disease Improving Global Outcomes; RRT, renal replacement therapy

Focusing on routine BCs, independent risk factors associated with BSIs were: V-A ECMO for graft failure after heart transplantation (OR 2.43, 95% CI [1.20; 4.92], *p* = 0.013) and BCs performed with ongoing antimicrobial therapy or renal replacement therapy (OR 2.15, 95% CI [1.08; 4.27], *p* = 0.029, and OR 2.05, 95% CI [1.10; 3.81], *p* = 0.008, respectively) (Table 6).

**Table 6:** Multivariate analysis of independent variables associated with positive routine blood culture

BSI characteristics	OR [95% CI]	*p*
**Primary graft failure**	2.43 [1.20; 4.92]	0.013
**Ongoing antimicrobial therapy**	2.15 [1.08; 4.27]	0.029
**Ongoing RRT**	2.05 [1.10; 3.81]	0.008

BSI, bloodstream infection; RRT, renal replacement therapy

**Fig. 3:**
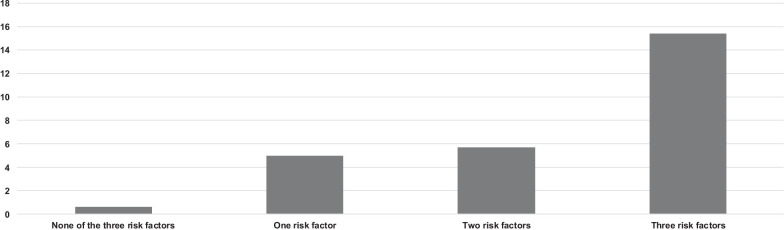
Occurrence of bloodstream infection diagnosis (%) with routine blood culture depending on risk factors

Figure 3 represents the occurrence of BSIs when BCs were collected systematically according to the presence of none or at least one of the three independent variables described above. In case of the absence of all these three conditions, only two BSIs from routine BCs were positive and would not have been caught up by on-demand BCs sampling. On the contrary, 15% of routine BCs revealed BSIs in the presence of all the three conditions. Test performance of the routine BCs strategy comparing the existence of at least one risk factor with the absence of risk factor was 96%, 30%, 5%, and 99%, for sensitivity, specificity, positive predictive value, and negative predictive value, respectively.

## Discussion

In this 4-year series of 150 consecutive V-A ECMO, BSIs were observed in one-third of patients, with a rate of 34.5 BSI/1.000 days of V-A ECMO support. Routine BCs identified significantly less BSIs than on-demand BCs and led to a high proportion of contaminations with subsequent inappropriate antimicrobial therapy. In addition, performing routine BCs were negatively associated with BSIs diagnosis. However, one-third of BSIs would have been missed by a policy restricted to on-demand BCs. This argues for better selecting conditions of routine BCs prescription on V-A ECMO. Indeed, this study highlighted three independent risk factors for BCs positivity when routine BCs were performed: patient with V-A ECMO for graft failure after heart transplantation, BCs collected with ongoing antimicrobial therapy or renal replacement therapy.

A recent study of 220 V-A ECMO reported an incidence of nosocomial infections of 64%, with ventilator-associated pneumonia and BSI being the most frequent (55% and 18%, respectively) [2]. The specific burden of infectious complications is hard to evaluate, because the most severe patients die early, while the survivors experience prolonged ECMO support and ICU length of stay. For instance, V-A ECMO support duration, ICU, and hospital length of stay were significantly longer in patients with at least one BSI, but this link reflects a longer vulnerability period, prone to septic complications. This precludes comparison of the mortality of infected versus non-infected patients, in addition to discrepancies between definitions and differences of case mix between studies.

A few studies focused on the incidence of BSI on ECMO support. Unfortunately, mixture of veno-venous (V-V) and V-A ECMO, various indications of support (from acute respiratory distress syndrome to refractory cardiogenic shock), and various types of population (pediatric for the most of studies, or a mix of adult and children) creates heterogeneity [24–28]. In addition, prophylactic anti-infective strategies and BCs contaminants varied between studies. Similarly, while the ELSO Infectious Disease Task Force does not recommend antibiotic prophylaxis on ECMO, 74% of centers reported performing it [29, 30]. The risk of BSIs and the well-established consequence of delayed or inappropriate antimicrobial therapy must be balanced with the negative impact of antibiotics on microbiota and subsequent infections [31].

Overall, a recent review (in pediatric and adult population with V-V and V-A ECMO) found a BSIs prevalence ranging from 3 to 18% and a incidence range from 3 to 31 episodes per 1.000 ECMO-days [3]. Regarding our BSIs rate, this large variation from a factor 1 to 10 prevents from drawing an “usual” rate of BSIs on ECMO. Our high rate of BSIs may be explained by (1) our daily routine BCs sampling protocol, (2) the inclusion of post-cardiac arrest patients who develop high rate of infectious complications, and (3) the extent of the study period up to 5 days after V-A ECMO withdrawal.

To deal with the challenging issue of diagnosis of infection on ECMO, routine BCs sampling is sometimes advocated but is still debated [6, 32]. According to ELSO surveys, between 34 and 49% of centers reported performing routine BCs, with variable intervals [8, 30]. As far as we are aware, this is the first study focusing on the interest and impact of a policy of routine BCs on V-A ECMO.

Multiple and non-clinically oriented routine BCs could increase the probability of diagnosing paucisymptomatic BSIs, but also the risk of positivity with contaminants. Our contamination rate was 2.5%; a rate within previously reported contamination ranges from 0.6 to 6% [33]. This raises several points of concern. First, the isolation of positive BC with potential contaminants generates an additional cost of hospitalization of around $8000 per patient, an increase of antibiotic prescription, and length of stay [34, 35]. It has been demonstrated than half of patients with contaminated BCs were inappropriately treated with antibiotics, one-third receiving vancomycin [17]. In our unit, antimicrobial therapy was deemed a posteriori inadequate in only 14% of cases, thanks to a rigorous anti-antimicrobial therapy policy and daily rounds with infectious diseases specialists. This may counterbalance our high rate of BSIs and contaminated BCs. Second, collecting 20 ml of blood per BC on the top of others daily blood samples exposes to progressive anemia [36]. On the contrary, a policy of reduction of blood laboratory tests reduced red blood cell transfusion, hospitalization costs, without impacting ICU outcome [37].

Our results question the optimal policy of BC practices. Our results could mean that BSIs are often symptomatic in this population, with either worsening shock or clinical/radiological signs of sepsis. Alternatively, physicians may have not prescribed on-demand BCs, knowing the fact that routine BCs had been sampled a few hours before. This subjective point may have lowered the diagnosis yield of on-demand BCs. Aiming to identify the clinical settings that increase the putative contribution of systematic BCs, we highlighted three risk factors for BC positivity. ECMO support after cardiac transplantation raises the question of performing systematic daily BC, as immunodepression increases the risk of BSI, and thus, the suitability of routine BC. Similarly, ongoing renal replacement therapy denotes higher severity and thus higher risk of nosocomial infections. Alternatively, the need for intravascular dialysis catheter may be a supplementary BSI risk factor. At least, BC sampling during ongoing antimicrobial therapy may reveal creeping bacteremia that are uncompleted treated by antibiotics. Overall, we propose a mitigated strategy, i.e., performing daily BC in adult patients on V-A ECMO with risk factor. This approach could be an acceptable compromise between an aggressive BC collection policy leading to an excess of contaminant identification and blood depletion, and a sparing policy missing clinically relevant BSI.

Our work presents limitations. First, in the absence of consensual definition of contaminated BCs in the present particular clinical setting, the CDC definition was applied [18, 38]. Second, our study population mixed patients with post-cardiotomy shock, acute heart failure, and refractory cardiac arrest. The latter is more prone to infectious complications, notably BSI [39]. Third, in case of death on V-A ECMO support, BC may not have been sampled the day of death. Fourth, the retrospective design of this study did not allow us to assess formally that all BCs taken between 4 and 7 a.m. were all routine sampling. Fifth, the reason of performing on-demand BC was not collected. We also acknowledge that providers who know their patients will receive daily BCs in the morning might be less likely to order on-demand cultures, or consider that the routine BCs were enough for diagnosing infection, even in the setting of new signs or symptoms. In addition, ongoing antimicrobial therapy was analyzed as a yes/no variable, but it may have different impact according to the duration of treatment. Also, we did not extract biological variables at each BC sampling but only those at V-A ECMO implantation, i.e., at day 0. Similarly, location and number of intravascular catheters were not collected, whereas they are per se a major risk factor for BSI. Sixth, BC was considered as the gold-standard of BSI diagnostic method. However, BC detect only culturable bacteria. Innovative techniques like bacterial DNA detection in rapid molecular assays such as PCR or more recently metagenomic next-generation sequencing (mNGS) test using cell-free DNA from blood could be promising tools for diagnosis supplementary BSI [40, 41]. Finally, the temporal window of BCs collection is debatable. Indeed, the time frame of ECMO-related BSIs advocated by some authors is from the day of V-A ECMO implantation up to two days after withdrawal [2, 42]. However, risk of delayed V-A ECMO-related BSIs persists several days after withdrawal. Our results confirm this recent finding, arguing for large BCs sampling after V-A ECMO withdrawal [43].

## Conclusion

We describe for the first time the consequences of BC practices policy in an adult population supported by V-A ECMO. Whereas routine daily BCs were less clinically relevant than on-demand BCs and lead to a significant proportion of inappropriate antimicrobial therapy, a restricting policy of BCs sampling misses a significant proportion of BSIs. We suggest daily BCs sampling on V-A ECMO support when easy-to-identify positive BCs risk factors are present. Further prospective studies should test these criteria.

## Data Availability

Data and materials are available on request to the corresponding author.

## Abbreviations

BC: Blood culture
BMI: Body mass index
BSI: Bloodstream infection
CERAR: Comité d’Ethique pour la Recherche en Anesthésie-Réanimation
CoNS: Coagulase-negative staphylococci
COPD: Chronic obstructive pulmonary disease
ELSO: Extracorporeal Life Support Organization
ICU: Intensive care unit
KDIGO: Kidney Disease Improving Global Outcomes
RRT: Renal replacement therapy
SAPS II: Simplified Acute Physiology Score 2
SOFA: Sepsis-related organ failure assessment
V-A ECMO: Veno-arterial extracorporeal membrane oxygenation
V-V ECMO: Veno-venous extracorporeal membrane oxygenation
